# Taxonomic novelties in Amaryllidaceae from the Department of Ancash, Peru, and a new combination in *Clinanthus*

**DOI:** 10.3897/phytokeys.131.36160

**Published:** 2019-09-16

**Authors:** Alan W. Meerow, Asunción Cano

**Affiliations:** 1 USDA-ARS-SHRS, National Germplasm Repository, 13601 Old Cutler Road, Miami, Florida 33158, USA USDA-ARS-SHRS, National Germplasm Repository Miami United States of America; 2 Montgomery Botanical Center, 11901 Old Cutler Road, Coral Gables, Florida 33156, USA Montgomery Botanical Center Coral Gables United States of America; 3 Laboratorio de Floristica, Departamento de Dicotiledoneas, Museo de Historia Natural, Universidad Nacional Mayor de San Marcos, Av. Arenales 1256, Lima 11, Perú Universidad Nacional Mayor de San Marcos Lima Peru

**Keywords:** Amaryllidaceae, Andean biodiversity, Clinantheae, Hymenocallideae, *
Ismene
*, monocots, Neotropical flora, taxonomy

## Abstract

*Clinanthus inflatus* (Amaryllidaceae) and *Ismene parviflora* are described from Ancash Department in Peru. The flower of *C. inflatus* is urceolate, and resembles that of *Urceolina* (Amaryllidaceae tr. Eucharideae), a unique morphology for the genus. *Ismene parviflora*, with its small, loosely formed, narrowly funnelform-tubular perigone with a ventricose limb, appears to have some affinity to subgen. Pseudostenomesson and may represent an intermediate form between the former and species of subgen. Ismene. *Stenomesson rubrum* is transferred to *Clinanthus* as *C. ruber* on the basis of its narrowly lorate leaf morphology.

## Abstract (es)

*Clinanthus inflatus* (Amaryllidaceae) e *Ismene parviflora* se describen del departamento de Ancash en el Perú. La flor de *C. inflatus* es urceolada, y se asemeja a la de *Urceolina* (Amaryllidaceae tr. Eucharideae), una morfología única para el género. *Ismene parviflora*, con su forma suelta, perigonio angostamente funeliforme-tubular con una limbo ventricoso, parece tener alguna afinidad al subgen. Pseudostenomesson y puede representar una forma intermedia entre el primero y las especie de subgen. Ismene. *Stenomesson rubrum* se transfiere a *Clinanthus* como *C. ruber*, sobre la base de su morfología de hoja acintada estrecha.

## Introduction

Amaryllidaceae is a cosmopolitan family of geophytic herbs (Meerow and Snijman 1998), comprised of three subfamilies: Agapanthoideae Endl., Allioideae Herb., and Amaryllidoideae (Chase et al., 2009), with the largest number of genera placed in Amaryllidoideae (Meerow and Snijman 1998). The subfamily has three main areas of diversity: southern Africa, Eurasia, and the Americas (Meerow and Snijman 1998; Meerow et al. 1999; Meerow et al. 2000; Meerow and Snijman 2001; Meerow and Clayton 2004; Meerow et al. 2006). Phylogenetic analyses of DNA sequences have resolved close cladistic relationships along biogeographic lines (Meerow et al. 1999; Meerow et al. 2000).

Meerow et al. (1999) demonstrated with multiple plastid sequences that the endemic American genera were sister to the Eurasian clade, and further that the American clade was comprised of two sub-clades, the so-called Hippeastroid and Andean tetraploid clades (Meerow 2010; Meerow et al. 2000). The Hippeastroids consist of two tribes, Hippeastreae and Griffineae Rav. (García et al. 2017; Garcia et al. 2014; Meerow et al. 2000). The Andean tetraploid clade contains four monophyletic tribes: Eucharideae Hutch., Eustephieae Hutch., Clinantheae Meerow, and Hymenocallideae Meerow (Meerow 2010; Meerow et al. 2000). The new species described in this paper are members of Clinantheae and Hymenocallideae, respectively.

*Clinanthus* Herb. was segregated from *Stenomesson* Herb. by Meerow et al. (2000), who demonstrated with nrDNA ITS sequences that the latter was polyphyletic. There are 15–20 species in the genus, which has never been monographed (León et al. 2006; unpubl. herbarium data). Peru is its center of diversity, and the genus occurs from southern Ecuador to northern Chile (Leiva and Meerow 2016). There are still novelties in the genus that await description (A. Meerow, pers. observ.). The genus is particularly diverse in northern Peru, and the complex orogeny of the Andes in Peru seems to have functioned as a species pump for the genus (Meerow 2010). The species are primarily found above 2000 m (León et al. 2006; unpubl. herbarium data), but several have colonized the Peru Current-cooled hills (*lomas*) of the Peruvian Pacific coast at much lower elevation (León et al. 2006; unpubl. herbarium data), in some cases occurring both there and in the Andes [e.g., *C. coccineus* (Ruiz & Pav.) Meerow]. One species, *C. humilis* (Herb.) Meerow, which retains the ovary inside the bulb until shortly before seed ripening (Herbert 1839; Baker 1871), reaches elevations above 4000 m (León et al. 2006; unpubl. herbarium data). Many are local endemics known only from the type localities (León et al. 2006). Photos and a specimen at USM sent to the first author for identification have been determined to represent an undescribed species in the genus, with unique floral morphology. We describe it here as *Clinanthus inflatus*. We also make a new combination in the genus *Clinanthus* for the species hitherto known as *Stenomesson rubrum* Herb. This species is transferred to *Clinanthus* based on its narrowly lorate leaf morphology.

*Ismene* Salisb., along with *Leptochiton* Sealy, are the central Andean components of tribe Hymenocallideae, with probably no more than 10 species (A. Meerow, unpubl. data). Many are also local endemics, primarily in Peru, but extending to Ecuador and Bolivia (Meerow unpubl. data). A number of years ago the first author received photos and a specimen on loan from the second author for identification. The plant was determined to be an undescribed *Ismene* species, unique for both the small size of the flowers and their morphology, strikingly divergent from the rest of the species in the genus. We describe it herein as *Ismene parviflora*.

## Material and methods

No specimens matching the new species other than the holotypes have been observed in herbarium collections in Peru, nor encountered by the first author in collections examined over the past 40 years at F, GB, K, MO, and NY. Herbarium acronyms follow Thiers (2019). Descriptions are based on holotypic material. Terminology used for the morphological descriptions follow Stearn (2004), Meerow and Snijman (1998), and Radford et al. (1974), with minor modifications. Photographic plates were composed with Corel Paint Shop Pro 2018 (Corel Corp., Ottawa, Ontario, Canada) from photos taken or received by both authors. The distribution map was also created with Corel Paint Shop Pro 2018 using license free maps of Peru (https://i.pinimg.com/736x/0c/9e/40/0c9e4008594c2228041624520f483f89--volunteers-the-south.jpg) and South America (https://i.pinimg.com/originals/67/83/ee/6783eeaff49338e5e4ae05c21d4b8312.jpg), both downloaded from Pinterest.

## Results

### Taxonomic treatment

#### Clinanthus inflatus

Taxon classificationPlantaeAsparagalesAmaryllidaceae

Meerow & A.Cano sp. nov.

B50F189AB6DD5067AF05802B08D63BA7

urn:lsid:ipni.org:names:60479369-2

Figs 1A–C , 2

##### Diagnosis.

This species differs from all known species of *Clinanthus* by the uniquely inflated perigone, the shape and coloration of which is reminiscent of the genus *Urceolina* Rchb. (Eucharideae) or superficially certain species of Andean vaccinioid Ericaceae.

##### Type.

PERU. Ancash: Prov. Recuay, Dist. Cotaparaco, Sector Santa Cruz, 2450 m, 6 Mar 2007. *M. Morales & E. Jara 767* (holotype: USM!).

##### Description.

**Figure 1.**
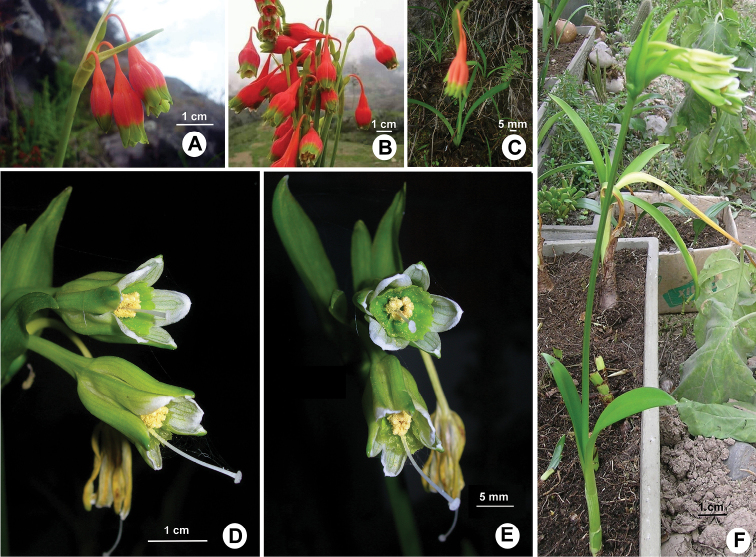
**A–C***Clinanthus inflatus*. Photos by anonymous source, used with permission. **A** Inflorescence in habitat **B** bouquet of cut inflorescences **C** habit in nature **D–F***Ismene parviflora*, photos by Asunción Cano **D–E** flowers **F** habit in cultivation.

Small terrestrial geophytic herb (Fig. 1A); bulb small, globose-ellipsoidal, ca. 2 cm diam., prolonged into neck 7.5–8 × 60–70 mm. Leaves 2–3, sessile, narrowly lorate, 4–6 × 90–110 mm, slightly canaliculate, obtuse at apex, with conspicuous midrib, glabrous, green, synanthous. Inflorescence pseudo-umbellate, borne at apex of naked scape; scape cylindrical, solid, 150–200 mm long (as observed), terminated by 2 spathe bracts, bracts green, ovate lanceolate. Flowers 2–3, pendulous, 35–40 mm long; pedicels slender, 15–20 mm long at anthesis; perigone consisting of 6 tepals in 2 whorls, fused into a tube that is narrowly cylindrical in proximal 15–17 mm and arcuate proximally, abruptly dilated distally to ca. 8 mm at throat; limb urceolate, abruptly inflated to ca. 33 mm in diam., then slightly constricted in distal 10 mm; tepals 6, bright orange-red proximally, concrescent, green in distal 10 mm with yellowish green margins, glabrous; outer tepals elliptical, 5–6 × 13–14 mm, apiculate; inner tepals elliptical, 5.5–6.5 × 12–13 mm, minutely apiculate. Stamens 6, basally connate into cylindrical staminal cup or corona, 7.4–7.6 × 11.0–11.5 mm, reaching to ca. 2 mm from the apex of the limb, salmon proximally, prominently 6-lobed, the lobes white distally and coarsely dentate along their edge, ca. 2 × 3 mm; free filaments short, inserted at sinus between the lobes; anthers grouped in the center of the flower (but not connivent), ca. 3 mm long, linear, dorsifixed, versatile, longitudinally dehiscent; pollen yellow. Style ca. 45 mm long, exerted ca. 5 mm past apex of the limb, white; stigma capitate, papillate, white, 2–2.3 mm wide. Ovary inferior, green, oblong, ca. 5 × 10 mm, 3-loculed, placentation axile, ovules oblong, flattened, ca. 20 per locule, superposed. Capsule and seed not seen.

**Figure 2.**
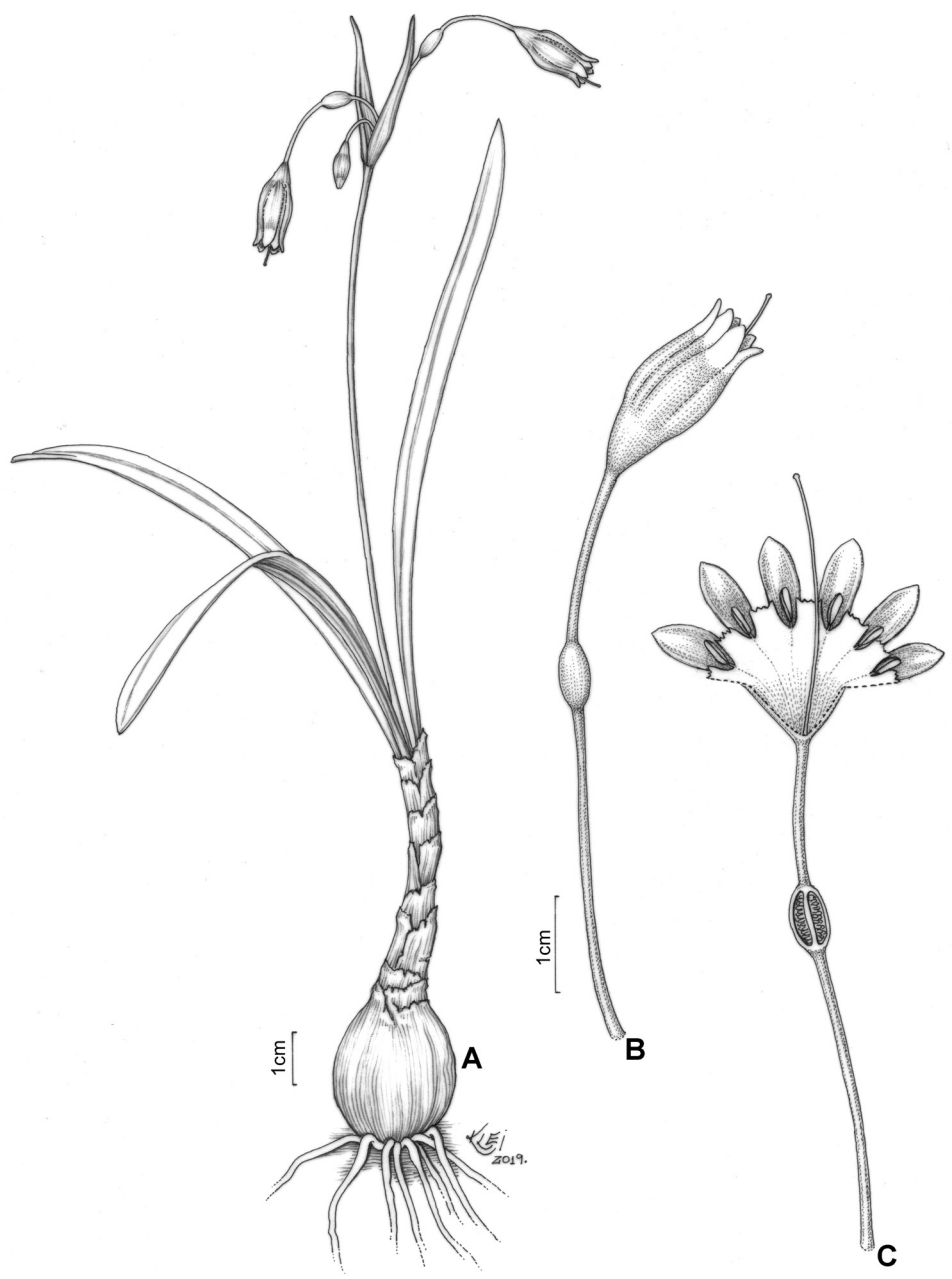
*Clinanthus inflatus*. **A** Habit **B** whole flower **C** whole flower cut open to show staminal corona and ovule number. Drawing by Klei Sousa.

##### Distribution and ecology.

**Figure 3.**
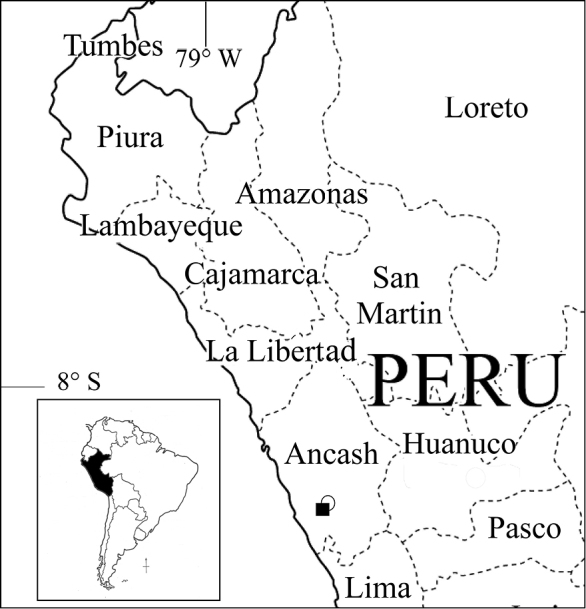
Map of northern Peru showing type localities of *Clinanthus inflatus* (white circle) and *Ismene parviflora* (black square). Inset: map of South America with Peru filled in black.

*Clinanthus inflatus* is known only from the type locality (Fig. 3), in seasonally dry vegetation.

##### Phenology.

Plants were collected in flower in March.

##### Etymology.

The specific epithet is from Latin and refers to the abruptly inflated perigone.

##### Preliminary conservation status.

Since nothing is known of the distribution of this species beyond the type locality, it is best to place it in the category Data Deficient (IUCN 2012, 2017). The type specimen label indicates that it was abundant at the collection site.

##### Notes.

The urceolate perigone of *C. inflatus* is yet another example of the convergent evolution that characterizes the tetraploid Andean lineages (Meerow 2010). It seems to have affinity with the subclade of *Clinanthus* (Meerow et al. 2000) that includes *C. campodensis* (Ravenna) Meerow, *C. humilis*, *C. recurvatus* (Ruiz & Pav.) Meerow, and *C. ruber* (Herb.) Meerow & A. Cano, all with leaves < 1 cm wide, and relatively small flowers.

#### Clinanthus ruber

Taxon classificationPlantaeAsparagalesAmaryllidaceae

(Herb.) Meerow & A.Cano comb. nov.

786E4583A722591892B6E8F603936009

urn:lsid:ipni.org:names:60479371-2

**Figure 4.**
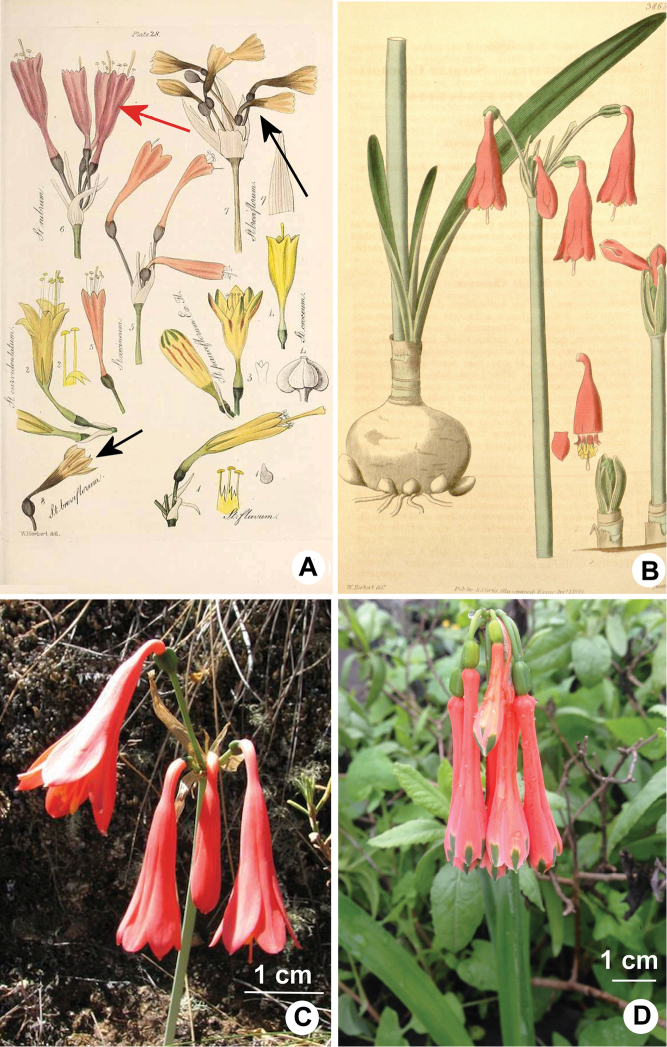
**A** Plate 28 from Herbert (1837) with *Stenomesson rubrum* Herb. illustrated (number 6, red arrow). *Stenomesson breviflorum* Herb. is illustrated in the same plate (numbers 7 & 8, black arrows), evidence that Herbert considered these two different species **B** Painting of *Coburgia coccinea* Herb. [= *Clinanthus ruber* (Herb.) Meerow & A. Cano] that accompanied the description by Herbert (1841)**C***Clinanthus ruber*, photographed in Peru. Photo by Oscar de Viveroscar **D***Clinanthus coccineus* (Ruiz & Pav.) Meerow, loma form, photographed in Lima Dept., Peru. Photo by Norton Cuba Melly.

Stenomesson rubrum Herb. Amaryllidaceae: 199; Pl. 28. 1837. Pancratium rubrum Pav. ex Steud. Nomencl. Bot. [Steudel], ed. 2. 2: 251. 1841. TYPE: Pavon s.n. (Holotype: FI!, isotype: BM!).
Coburgia coccinea Herb., Curtis’s Bot. Mag. 67: t. 3865. 1841, syn. nov., non Coburgia coccinea (Ruiz & Pav.) Herb., Edwards’ Bot. Reg. 28 (Misc.): 54 (1842). (Fig. 4B, D).

##### Notes.

*Pancratium rubrum* (Herb.) Pav. ex Steud. was not included in Ruiz and Pavon (1802), even though a specimen from their expedition (FI!, FI011974) is labelled as the type of *S. rubrum* Herb. The name was validated by von Steudel (1841). Ravenna (1978) believed that the type specimen represented *Stenomesson flavum* (Ruiz & Pav.) Herb., a species that was originally illustrated and described in Ruiz and Pavon (1802) as *Pancratium flavum* Ruiz & Pav. We believe that Ravenna (1978) was mistaken in this regard. The specimen has a single scape that bears only three flowers, and the stamens are barely if at all exerted from the perigone. *Stenomesson flavum* always has > 3 flowers and the stamens are exerted at least 1 cm from the limb.

Herbert (1837) illustrated *S. rubrum* (Fig. 4A), which he seemed to consider distinct from *Stenomesson coccineum* (Ruiz. & Pav.) Herb. [≡ *Clinanthus coccineus* (Ruiz. & Pav.) Meerow], a decision with which we agree. Herbert (1837) also surmised that errors were made regarding Ruiz and Pavon types and plates in regard to both species. Four years later, Herbert (1841) published *Coburgia coccinea* Herb. (Fig. 4B). He made no reference to either *S. rubrum* or *S. coccineum*, though the flower in the plate (Fig. 4B) looks very similar to the flower illustrated for *S. rubrum* (Fig. 4A). The well-illustrated leaf morphology in the plate undoubtedly places the plant in *Clinanthus*. One year later, Herbert (1842) assigned both *Pancratium coccineum* Ruiz [sic] and *Stenomesson coccineum* to synonomy with *Coburgia coccinea*. We thus regard *Coburgia coccinea* Herb. (1841) as distinct from *Coburgia coccinea* (Ruiz & Pav.) Herb. (1842), nom. illeg., but conspecific to *Clinanthus ruber*, and include it in the synonymy of the latter taxon here.

Ravenna (1978) diagnosed *Clinanthus coccineus* (Ruiz & Pav.) Meerow as belonging to his Stenomesson subgen. FulgitubaRavenna (1974), along with *Clinanthus incarnatus* (Kunth) Meerow, *C. microstephius* (Ravenna) Meerow, *C. variegatus* (Ruiz & Pav.) Meerow and other species, all of which have varying apical zones of green on the tepals. Recent collections in Peru (N. C. Melly, S. Leiva, pers. comm.) confirm this, with *Clinanthus coccineus* (Fig. 4D) found both in coastal *lomas* and in the northwestern Andean slopes. Ravenna (1978) seems to have considered *S. rubrum* a synonym for *S. breviflorum* Herb. We disagree with this assessment. Herbert (1837) illustrated both in the same plate (Fig. 4A). The type specimen of *S. breviflorum* (Maclean s.n. K!, K000322395 & 396) clearly bears pseudo-petiolate leaves, which confirms its place in *Stenomesson* rather than *Clinanthus*. *Clinanthus ruber* is fairly widespread in northern Peru, and can have red or pink flowers, but never more than five in all the material we have seen. It is variable in the degree to which the limb spreads from the throat of the perigone. The flowers are without any green apical zone, and bear relatively narrow leaves < 1 cm wide.

#### Ismene parviflora

Taxon classificationPlantaeAsparagalesAmaryllidaceae

Meerow & A.Cano sp. nov.

D6FF55168E585D7497F024041805E555

urn:lsid:ipni.org:names:60479370-2

Figs 1D–F , 5

##### Diagnosis.

*Ismene parviflora* differs from all other species of the genus by the small flowers (< 4 cm long), that are loosely funnelform, terminating with a ventricose limb, but not infundibular to the degree of *I. vargasii* and *I. morrisonii* (subgen. Pseudostenomesson Meerow), the latter species to which it approaches in size. All other species of Ismene have large, crateriform flowers (subgen. Ismene) or large, zygomorphic flowers (subgen. Elisena).

##### Type.

Perú, Ancash: Prov. Recuay, Dist. Cotaparaco, Sector Santa Cruz,, 2332 m, rocky slope with shrubs and silty soil, 6 Mar 2007. *M. Morales & E. Jara 767* (holotype: USM!).

##### Description.

Terrestrial geophytic plant from subterranean bulb. Bulb not seen. Leaves 2, oblanceolate-lorate, ca. 15 × 150–170 mm, bright green, glabrous, slightly canaliculate, obtuse, tightly sheathing proximally and forming a green aerial pseudostem 40–60 mm long, synanthous. Inflorescence pseudo-umbellate, borne on a naked scape; scape ancipitous, solid, 200–250 × ca. 18 mm, terminated by 2 ovate-lanceolate green bracts that persist at anthesis, over-topping flowers by several cm, bracts 50–55 × 7.5–9.6 mm, acute. Flowers 3–5, mostly perpendicular to scape, loosely funnelform-tubular, shortly pedicellate, pedicels 2–3 mm long; perigone 35–37 mm long, consisting of 6 tepals in two whorls, fused proximally into a tube that is cylindrical for proximal 20 mm, ca. 1.5 mm wide, then dilating to 3.5 mm at throat; tube curved proximally; limb slightly ventricose, apically ca. 10 mm wide, tepals loosely concrescent, not spreading significantly; outer tepals elliptical, concave, mostly green abaxially, white towards apex, adaxially white with green veins in distal ½–2/3, green below, 13.5–14.0 × 4.5–5.0 mm, apiculate; inner tepals ovate-elliptical, less concave than outer, 13.0–13.5 × 5.0–5.2 mm, minutely apiculate, colored like outer. Stamens 6, fused into green staminal cup 6.5–7.5 × 3.5–3.7 mm, cylindrical in proximal 6 mm, abruptly ampliate in its distal 1 mm to ca. 4 mm, coarsely and unevenly dentate at rim; free filaments inserted ca 2 mm below rim of cup, filiform, white, incurved, ca. 1 mm long; anthers oblong, closely appressed to each other but not connivant, 3.5–4.5 mm long, pollen yellow. Style exerted 10–15 mm past the limb apex, 40–45 mm long, white, filiform; stigma capitate, papillose, white, 2–3 mm wide. Ovary globose, ovules 1 or 2 per locule, basal. Capsule and seed not seen.

**Figure 5.**
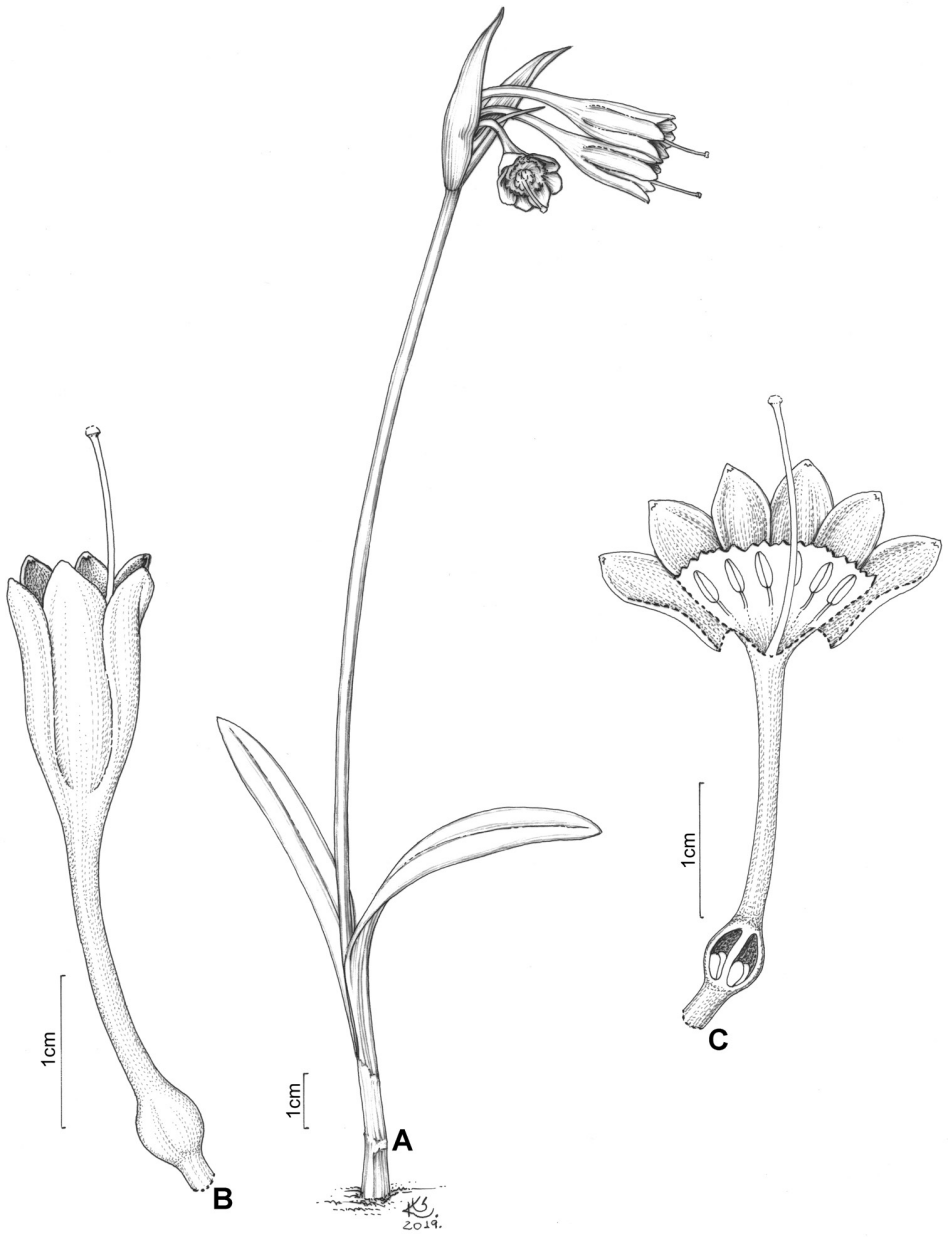
*Ismene parviflora*. Drawing by Klei Sousa. **A** Habit **B** whole flower **C** whole flower cut open to show staminal corona and ovule number.

##### Distribution and ecology.

*Ismene parviflora* is known only from the type locality (Fig. 3), in seasonally dry vegetation.

##### Phenology.

Plants were collected in flower in March.

##### Etymology.

The specific epithet is from Latin and refers to the small size of the flowers.

##### Preliminary conservation status.

Since nothing is known of the distribution of this species beyond the type locality, it is best to place it in the category Data Deficient (IUCN 2012, 2017). The type specimen label says it was abundant at the collection site.

##### Notes.

*Ismene parviflora* has greatest affinity morphologically to the two members of Ismene subgen. Pseudostenomesson (Velarde) Meerow, *I. vargasii* (Velarde) Gereau & Meerow and *I. morrisonii* (Vargas) Velarde, but the unique morphology of the new species make it difficult to assign *I. parviflora* to I. subgen. Pseudostenomesson with confidence at this time. Ravenna (1988) disagreed with the transfer by Velarde (1949) of *Stenomesson morrisonii* Vargas to *Pseudostenomesson* Velarde, but based on the same information (Vargas 1943), we believe it belongs with *I. vargasii* in I. subgen. Pseudostenomesson. In particular, the photo in Vargas (1943) shows the typical foliar morphology of *Ismene*, i.e. the aerial pseudostem formed by the tightly sheathing leaf bases. The two species of subgen. Pseudostenomesson are found in the Departments of Junin (*I. vargasii*) and Apurimac (*I. morrisonii*), and, unlike *I. parviflora*, have larger, fully infundibular, and completely green perigones.

## Supplementary Material

XML Treatment for Clinanthus inflatus

XML Treatment for Clinanthus ruber

XML Treatment for Ismene parviflora
